# Pelvic endometriosis: epidemiological and clinical profile prior to surgical treatment

**DOI:** 10.1590/1806-9282.20240746

**Published:** 2025-03-17

**Authors:** Danielle Costa Nazareth, Daniel Bier Caraça, Gabriela Guimarães Franco Ramos, Sergio Podgaec

**Affiliations:** 1 Hospital Israelita Albert Einstein – São Paulo (SP), Brazil.

**Keywords:** Endometriosis, Dysmenorrhea, Pelvic pain, Infertility, Video-assisted surgery

## Abstract

**OBJECTIVE:**

This study aims to analyze the epidemiological, sociodemographic, and clinical profile of women undergoing videolaparoscopic surgery for the treatment of pelvic endometriosis at the Hospital Israelita Albert Einstein.

**METHODS:**

A cross-sectional and descriptive study was conducted, including 110 patients who underwent surgery for endometriosis between January 2017 and December 2022. Sociodemographic information, personal history, symptomatology, radiological diagnosis, and surgical details were collected.

**RESULTS:**

The average age of the evaluated patients was 37.04 years. Most were white women (69.7%), married or in a stable union (57.8%), and had completed secondary or higher education (86.3%). Dysmenorrhea was the predominant complaint in 60% of cases, with symptom onset occurring mainly between 1 and 5 years (48.2% of the total). Postoperative complications were observed in 8.1% of participants (9 cases), while the main associated comorbidities were anxiety and depression, represented in 43.3% of cases (26 cases).

**CONCLUSION:**

Endometriosis presents as a condition that negatively impacts women's quality of life, generally diagnosed between the fourth and fifth decades of life. Hormonal treatment is sometimes insufficient to resolve symptoms, requiring surgical intervention for clinical improvement.

## INTRODUCTION

Endometriosis is a prevalent gynecological condition characterized by its chronic and benign nature^1,2^. Studies indicate that it affects between 10 and 15% of the female population of reproductive age. Its prevalence ranges from 2 to 11% in asymptomatic women, from 5 to 50% in women with infertility, and from 5 to 21% in women hospitalized due to pelvic pain^3,4^.

The disease has been classified into three types: peritoneal, ovarian, and deep. Peritoneal endometriosis is characterized by superficial implants on the peritoneum; ovarian endometriosis by superficial implants on the ovary (endometriomas); and deep endometriosis by lesions penetrating the retroperitoneal space or the walls of pelvic organs, with a depth of 5 mm or more^5^.

The heterogeneity of symptoms is notable, with manifestations including dysmenorrhea, acyclic pelvic pain, deep dyspareunia, intestinal and/or urinary alterations, infertility, or even being asymptomatic^6,7^. Although definitive confirmation is through surgical intervention, the combination of physical examination and imaging can also be effective for diagnosing the condition^3^.

Transvaginal ultrasound with bowel preparation and magnetic resonance imaging are the recommended complementary diagnostic exams. The management of cases varies according to certain criteria^8^.

Hormonal clinical treatment has shown effectiveness in controlling pelvic pain and should be the preferred option in the absence of absolute indications for surgery^2,9,10^.

Due to the heterogeneity of the clinical presentation, it is important to improve the identification of the characteristics of these patients. Thus, the objectives of this study were to describe the epidemiological and clinical aspects of endometriosis in patients with an indication for surgical treatment^2,3^.

## METHODS

This is a cross-sectional, descriptive, and retrospective study conducted with 110 patients who underwent surgical treatment for endometriosis at the Hospital Israelita Albert Einstein, referred by the "Cuidar program." The study period was from January 2017 to December 2022. The project was approved by the Human Research Ethics Committee and followed Resolution 466/2012 of the National Health Council.

Data collected included age, clinical presentation, obstetric history, associated comorbidities, diagnostic method for surgical planning, prior hormonal treatment, surgical data, and postoperative complications. Symptom intensity was assessed using the visual analog scale.

Data were analyzed using descriptive and inferential analyses, including descriptive statistics, frequencies, measures of central tendency and distribution, contingency analyses, and Mann-Whitney tests. The effect size was calculated and classified as proposed by Fritz et al. ^11^. The IBM^®^ SPSS^®^ software (Amos™; Version 23) was used for the analyses, with a significance level set at p<0.05 for all inferential analyses.

## RESULTS

Records of 110 patients who underwent surgical intervention for pelvic endometriosis treatment were examined. The demographic analysis revealed that the majority of patients were white women, totaling 62 patients (56.3%), 6 were black (5.4%), 5 were mixed race (4.5%), and 4 were Asian (3.6%). Additionally, approximately 30% of the study population did not have information about their race recorded in the medical records.

Most patients were married or in a stable relationship, representing 64 patients (58.1%). Meanwhile, 39 were single (35.4%) and 7 women were divorced (6.3%). Regarding education, the majority of patients, totaling 95 (86.3%), had completed high school or higher, while 23 of them (20.9%) had only completed elementary school. The mean age of the 110 analyzed patients was 37.04 years, with a standard deviation of 5.10 years.

Regarding symptoms, dysmenorrhea was the main complaint in 60% of cases (Table 1). Additionally, Table 2 shows all objectively reported symptoms and their frequency in the sample.

**Table 1:** Distribution of the main complaint presented by the patients.

Main complaint	Frequency (number of cases)	Frequency
Dysmenorrhea	66	60.0
Chronic pelvic pain	20	18.2
Infertility	9	8.2
Deep dyspareunia	7	6.4
Associated symptoms (more than one symptom described above)	7	6.4

**Table 2:** Distribution of symptoms reported by the patients.

Reported symptoms	Frequency (number of cases)	Frequency
Dysmenorrhea	78	70
Chronic pelvic pain	56	51
Infertility	11	10
Deep dyspareunia	65	59
Urinary symptoms	15	13
Intestinal symptoms	40	36

The assessment of pain intensity, performed using the visual analog scale from 0 to 10, revealed specific medians and interquartile ranges for each symptom (see Table 3).

**Table 3:** Description of visual analog scale values for each of the symptoms reported by the patients.

	Median	IQR	Min–max
Dysmenorrhea	8	2	0–10
Deep dyspareunia	6	4.75	0–10
Urinary symptoms	0	0	0–6
Intestinal symptoms	4	5	0–10
Chronic pelvic pain	6	7.5	0–10

IQR: interquartile range.

Regarding obstetric history, 44 participants (40% of the sample) were nulligravid, while the remaining 66 patients (60% of the sample) had a history of previous pregnancies, including vaginal delivery, cesarean section, and abortions.

Regarding the frequency of major comorbidities, the most common diseases were anxiety/depression, present in 26 patients, followed by hypertension in 9 patients and diabetes and thyroid diseases in 6 patients each.

All patients reported undergoing prior clinical treatment. Considering treatments lasting more than one year, 44 out of 110 mentioned prolonged use of combined hormonal methods in an attempt to alleviate symptoms. Progestogens, including oral, intrauterine device, and injectable formulations, were the main therapeutic options, used in 10, 12, and 2 cases, respectively. However, the need for more than one medication attempt, over a period of more than 1 year of use, was observed in 41 patients, of whom 36 (87.8%) used the levonorgestrel-releasing intrauterine device.

In the context of surgical planning, pelvic ultrasound and transvaginal ultrasound with bowel preparation were the most commonly used imaging methods, representing 58.2% of occurrences, followed by magnetic resonance imaging, used in 25.5% of cases.

Diagnostic laparoscopy was necessary in only 2 (2.7%) cases. In one case, the patient, with a wall endometrioma, also presented typical endometriosis symptoms, but imaging tests did not detect the condition. Laparoscopy revealed endometriosis foci on the peritoneum. In another case, laparoscopy was necessary due to the difficulty in demonstrating superficial foci by imaging tests. In 13.6% of cases, more than one diagnostic method was associated with surgical planning.

Of the procedures performed, the main affected sites confirmed by pathological examination were the uterosacral ligaments (58%), peritoneum (41%), ovaries (40%), rectosigmoid (37%), vaginal cuff (32%), and retrocervical region (22%). Regarding specific surgical procedures, highlights include the resection of lesions in uterosacral ligaments in 64 procedures, excision of lesions in the posterior cul-de-sac and vaginal cuff in 35 cases, oophoroplasty performed in 38 patients with ovarian endometriomas, with the majority on the left side (22 cases), and the approach to the rectosigmoid in 45 patients, which included segmental resection, discoid resection, and shaving.

Regarding postoperative complications, 8.1% of patients experienced adverse events (9 out of 110 cases): rectal anastomotic dehiscence (n=1), surgical wound infection (n=1), abdominal wall hematoma (n=1), pyelonephritis (n=2), rectal bleeding (n=1), deep vein thrombosis (n=1), and inadvertent bladder injury (n=1).

Regarding women with a history of infertility, of the nine patients who reported attempting pregnancy for more than 1 year after the surgical procedure, four conceived spontaneously, two conceived after in vitro fertilization, and three did not conceive upon return.

## DISCUSSION

A detailed analysis of the results of this study provides an understanding of the sociodemographic, epidemiological, and clinical profile of patients with endometriosis and surgical outcomes. The mean age of 37.04 years, with a standard deviation of 5.10 years, reflects the prevalence of this condition in women in the reproductive age, between the fourth and fifth decades of life, confirming literature data indicating a prevalence of 10–15% in the female population of reproductive age^3,4^.

Notably, there is a predominance of white women affected by endometriosis, covering 56.3% of cases, aligning with studies indicating rates of up to 97% for Caucasian women^4^. It is worth noting that although there are variations among different ethnic groups, these disparities do not reach statistical significance, suggesting that racial factors do not play a significant role as risk drivers for the disease^3,4,6^.

Regarding educational and socioeconomic profile, there is commonly a tendency for women with endometriosis to have higher levels of education^4^. However, despite our study including a heterogeneous sample of professionals, all had homogeneous access to the institutional healthcare system.

Regarding symptoms, dysmenorrhea was the most reported (60.0%). However, it was possible to identify an association between endometriosis and other frequently cited symptoms, such as deep dyspareunia, chronic pelvic pain, cyclic urinary, and intestinal symptoms^3,6^. It is worth noting that since the evaluated cases are from a reference service, where we operate all cases of the study, the high prevalence of disabling symptoms may be explained by this bias of patient selection.

In the context of intestinal endometriosis, this condition affects between 6 and 30% of women with deep endometriosis, manifesting as abdominal pain, constipation, discomfort during bowel movements, bleeding, or, more rarely, intestinal obstruction^2,3^.

In our study, we analyzed a group of 45 women with this type of involvement, with different approaches depending on the size of the lesion, with segmental resection for lesions larger than 3 cm or multiple, discoid resection for those smaller than 3 cm affecting the rectal muscle layer and shaving for more superficial lesions^2^.

Regarding previous treatment, combined oral contraceptives (COCs) were widely mentioned for attempting to control the disease, reflecting its common application as a conservative approach in many healthcare centers.

In a controlled study comparing COC to the use of GnRH analogs (GnRHa), equivalent efficacy was observed in reducing pelvic pain associated with endometriosis. However, continuous use of COC, especially in patients with predominant dysmenorrhea, provided superior short-term benefits, while the use of the analog was more associated with side effects^10^.

Still, regarding conservative treatment, other routes of progestogen administration, such as the levonorgestrel-releasing intrauterine system (LNG-IUS), have been shown to be effective in pain control, presenting a lower incidence of side effects, as found in a prospective, randomized, controlled study^9^.

Regarding surgical treatment, concerning the approach to intestinal lesions, although surgical resection is widely recognized as the most effective method for treating intestinal endometriosis, its indication remains debated due to the risks involved^2,6^.

In line with our research, we observed a low incidence of intestinal complications, with only one case of rectosigmoid anastomotic dehiscence and one episode of rectal bleeding among the procedures performed.

Still, regarding surgical procedures, studies point out the importance of removing the wall of ovarian endometriomas, rather than just aspirating them, due to the recurrence rates associated with the latter technique^1,12^.

A randomized study emphasized the benefits of cystectomy (capsule removal) over fenestration with coagulation, demonstrating lower symptom recurrence (15.8 vs. 56.7%) and the need for surgical reintervention (5.8 vs. 22.9%) after 24 months^1^. Our results show that 86% of cases of ovarian endometriomas underwent oophoroplasty, corroborating the effectiveness of this approach in clinical practice.

Another important association identified in the present study was the prevalence of psychiatric comorbidities in the studied population, where 26 patients reported a diagnosis of anxiety and/or depression. A recent study published by Wang et al., which included 100,770 participants in Taiwan between 2000 and 2015, revealed that patients with endometriosis have a 2.131 times higher risk of developing mental disorders compared to those without endometriosis^13^.

Donatti et al. investigated the relationship between coping strategies, depression, stress, and pain in 171 women with endometriosis, highlighting that positive coping strategies were associated with lower depression and better stress adaptation, as well as correlating with lower pain intensity^14^. Another study by the same author reviewed research that applied cognitive-behavioral therapy in patients with endometriosis and chronic pelvic pain, finding positive and lasting effects on pain reduction and psychological well-being, emphasizing the need for more specific intervention protocols^15^.

Finally, pelvic endometriosis is associated with difficulty in conception, as evidenced by several studies^4,11,16^. About 20–50% of women with difficulty conceiving are diagnosed with endometriosis, while between 30 and 50% of women with endometriosis face fertility problems^16^. The low incidence of fertility problems in our study may be explained by the fact that many participants were in the age range between the fourth and fifth decades of life and also by the use of contraceptive methods to control the disease.

## CONCLUSION

In this study, we investigated endometriosis in a specific sample of patients undergoing perioperative assessment at a reference hospital. We identified that the diagnosis of endometriosis predominantly occurred in the fourth decade of life, with symptoms of dysmenorrhea and pelvic pain being the most common. We highlight as a positive aspect the successful application of a minimally invasive surgical approach, even in cases of extensive disease.

These results suggest a promising perspective for the treatment of these patients. However, it is important to consider the limitations of the study. The patients were selected from the hospital staff and specifically referred to gynecological surgeons, which may have influenced the inclination toward surgical treatment. The lack of postoperative follow-up data on the patients also limits the interpretation of the results. Moreover, the absence of a control group in the descriptive study restricts the generalization of the findings.
